# Harm reduction and knowledge exchange—a qualitative analysis of drug-related Internet discussion forums

**DOI:** 10.1186/1477-7517-11-25

**Published:** 2014-09-08

**Authors:** Christophe Soussan, Anette Kjellgren

**Affiliations:** 1 Department of Psychology, Karlstad University, SE-651 88 Karlstad, Sweden

**Keywords:** Novel psychoactive substances, Legal highs, Research chemicals, Internet drugs, Harm reduction, Internet forum, Drug use

## Abstract

**Background:**

Novel psychoactive substances (NPS) are continuously and increasingly appearing on the international drug market. Global Internet forums are a publicly available reality where users anonymously discuss and share information about NPS. The aim of this study was to explore and characterize the discussions about NPS on international Internet forums.

**Methods:**

The most post-frequent NPS discussions were collected from three “leading edge” international Internet forums. A total of 13,082 posts from 60 threads of discussion were systematically examined and interpreted to reveal recurring topics and patterns. Each thread was coded with emerging topics and supporting quotations from the data set. Eventually, codes with coherent meaning were arranged into 51 broader categories of abstraction, which were combined into four overarching themes.

**Results:**

Four themes emerged during the analysis: (1) *uncovering the substance facts*, (2) *dosage and administration*, (3) *subjectively experienced effects*, and (4) *support and safety*. The first theme dealt primarily with substance identification, pharmacology, and assessed not only purity but also legal status and acquisition. The second theme focused on administration techniques, dose recommendations, technical talk about equipment, and preferred settings for drug use. The third theme involved a multitude of self-reported experiences, in which many different aspects of intoxication were depicted in great detail. The users emphasized both positive and negative experiences. The last theme incorporated the efforts of the communities to prevent and minimize harm by sharing information about potential risks of the harmful effects or contraindications of a substance. Also, online support and guidance were given to intoxicated persons who experienced bad or fearful reactions.

**Conclusions:**

The findings showed that the discussions were characterized by a social process in which users supported each other and exchanged an extensive and cumulative amount of knowledge about NPS and how to use them safely. Although this publicly available knowledge could entail an increase in drug use, the main characteristics of the discussions in general were a concern for safety and harm reduction, not for recruiting new users. Drug-related Internet forums could be used as a location for drug prevention, as well as a source of information for further research about NPS.

## Background

A profound shift in the market for recreational drugs has occurred. The availability and number of unregulated novel psychoactive substances (NPS) have continuously increased and expanded into a global phenomenon [1-3]. For four consecutive years, the European Union early warning system on drugs has detected a record high and growing number of new substances and online vendors. In total, 280 substances, including stimulants, synthetic cannabinoids, hallucinogens, dissociatives, and sedatives, are currently being monitored by the European Monitoring Centre for Drugs and Drug Addiction (EMCDDA). However, NPS prevalence is somewhat uncertain since the increase of substances could reflect an improved capacity for detection and monitoring [4]. Moreover, the limited number of demographic studies is often based on different substances or nonrepresentative samples and shows varying results across populations [5-7]. A survey [5] investigating the use of NPS among youth in Europe found that an average of 5% had experience of use, a number that differed significantly among nations. Most sources indicate that users are primarily young males [8,9], although a recent study [10] reported that middle-aged adults constituted a quarter of the sample.

NPS, also referred to as “legal highs”, “designer drugs”, or “research chemicals”, often mimic the effects of corresponding illicit drugs and are intentionally produced to circumvent existing drug laws [11]. Legal responses are to some extent ineffective, partly because clandestine chemists quickly adapt to and exploit new legislations by marketing substances with slight molecular deviation but preserved psychoactive effect [12-14]. In addition, NPS are often sold surreptitiously as, for example, “bath salt” or “plant food” and labeled “not for human consumption” in order to avoid legislative attention. Furthermore, the use of the Internet as a marketplace, rather than the streets, is believed to impede regulatory actions and promote availability [1].

There are very limited information and published data on NPS available to either users or health-care personnel [15,16]. These substances have rarely or never been subjected to studies on humans or animals, which make them highly unpredictable. Long-term effects, dependency potential, toxicological risks, or possible contraindications are largely unknown. In addition, content declaration or warnings about side effects or hazardous substance interactions are mostly lacking [17]. In order to provide knowledge of these substances, and patterns of human use, data from other sources like the Internet have been suggested and utilized for scientific studies [16,18-20]. For example, self-reports posted on public Internet forums have been analyzed to investigate the characteristics of experience with substances as 4-HO-MET, MXE, and “Spice” [21-23]. Although anecdotal data from the Internet have been claimed to be biased or unreliable [16,19], our recent NPS study [24] based on Internet discussion forum data showed that the results were highly congruent with the findings of studies based on clinical data.

Regardless of the degree of validity, publicly available content on the Internet is an undeniable reality, which remains the major source of information for youths with sensitive or health- and drug-related issues of concern [5,25,26]. Internet discussion forums provide a global and anonymous environment in which sharing of drug-related information is a prominent feature. Other documented forum characteristics include social cohesion and support, as well as a focus on harm reduction [27,28]. Another study [21] has also emphasized the social togetherness among closed groups of NPS users, which highlights a need to share and discuss with peers. Hence, there are strong reasons to further investigate drug-related Internet communities and the ongoing discussions in greater depth. It is important to examine what users are talking about explicitly, as well as investigating any implicit features of the discussions in general, not only to understand the reality facing young people online but also to examine the possibilities for future research and prevention strategies.

The aim of this study was to explore, define, and characterize the discussions about novel psychoactive substances on international Internet forums.

## Methods

### Data collection

The data were retrieved from the “leading edge” Internet forums, as presented by the Psychonaut Web Mapping Project [19], which identified the key online resources for the study of NPS. Three internationally oriented discussion forums with open access were found by Deluca et al. [19]: bluelight.org, drugs-forum.com, and legalhighsforum.com. No scientific or anecdotal evidence that contradicts the status of these sites as “leading edge” was found. Therefore, all three were considered up-to-date and included as source of data in the present study.

The 20 most recent threads of discussion in each subcategory of each forum were collected into a list (one for each forum), containing thread title and number of posts. Threads unrelated to NPS were removed. Each list was sorted by number of posts to find the threads most discussed in each forum. We labeled these threads “post-frequent” for future reference. The top 20 threads from each forum (a total of 60) were selected for further investigation and analysis. The total number of posts from all three forums was 13,082. All collected posts were in the English language. The gathering of data took place during October 2013, and the latest retrieved post was written October 22.

### Analysis

The 60 discussion threads were analyzed individually by reading all posts in each of them in pursuit of recurrent topics of discussion. The analysis process was characterized by openness and a bias-free attitude and was undertaken through a systematic exploration of discussion topics and patterns in each thread. More specifically, each post was examined and interpreted for its underlying topic. Every time a new topic emerged in each specific thread, a code was created and related to that thread. For example, the thread titled “*Big n Dandy 2-FA (2-fluoroamphetamine) thread*” contained the following post: “*I used this substance for the first time and experienced chest pains and aches all over the body*”, which was coded as “*Sharing experienced side effects*”. Furthermore, the thread titled “*Dose - The true Methylone dosage?*” contained the post “*My recommendation is wrapping 150-200 mg of Methylone in a rolling paper and ingest it on empty stomach*”, which was coded as “*Dosage suggestion*” and “*Administration suggestion*”. The coding was done manually in a Word document. Eventually, all 60 discussion threads had a set of codes and multiple accompanying quotations to support the codes. Also, the codes were repeatedly checked for consistency through confirmation by comparison with other posts on the same topic in the same thread. On occasion, previously coded threads were re-read as new topics emerged in other threads, which could have been missed. At one point, the codes were saturated, meaning no new topics emerged, which was interpreted as indicating that the largest part of discussion topics was covered by the analysis. Next, codes with coherent meaning were arranged into 51 categories (see Table 1). Finally, all the categories were combined into four overarching themes that characterized the discussions: (1) *uncovering the substance facts*, (2) *dosage and administration*, (3) *subjectively experienced effects*, and (4) *support and safety*.

**Table 1:** The 51 categorized topics and the four overarching themes characterizing the discussions

**Themes**	**Topics**
Uncovering the substance facts	Brands
	Service
	Contraindications
	Content speculation
	Batches
	Vendors
	Reviews and evaluations
	Acquisition
	Specific substances
	Availability
	Toxicity
	Quality
	Legal status
	Price
	Tolerance and cross-tolerance
	Pharmacology
	Comparing substances
Dosage and administration	Supplements
	Purpose and intention
	Combinations
	Administration
	Expectations
	Settings
	Mood and state
	Dosage
	Do it yourself
	Recreation
Subjectively experienced effects	Initial effects
	Aftereffects
	Side effects
	Positive effects
	Physiological
	Emotional
	Cognitive
	Duration
	Potency
	Evaluating effects
	Experiences
	Insight
	Comparing effects
Support and safety	Coping
	Harm reduction
	Caution
	Acute help
	Risks
	Warnings
	Recovery reports
	Addiction
	Support
	Advice

### Ethical considerations

Our research involved the collection and analysis of already existing information that were published on public Internet forums. No terms of access or special permissions restricted the discussions from public access. A discrete and observational approach was undertaken, and no interactions or interventions with forum discussions or members were made. The information available was therefore considered to be an observation of public behavior online, in compliance with the ethical guidelines and recommendations provided by SACHRP [29]. In order to further strengthen the anonymity of the forum members, we stripped the data set from user aliases and URLs. Also, a careful assessment of anonymity and search engine visibility for every presented quotation was undertaken before publishing. Certain quotation details have been altered in order to further protect the users’ anonymity.

## Results

The analysis of post-frequent and drug-related discussions on international Internet forums generated 51 categorized topics based on 13,082 user posts, which were combined into four overarching themes of discussion: (1) *uncovering the substance facts*, (2) *dosage and administration*, (3) *subjectively experienced effects*, and (4) *support and safety*. The themes are presented below with some representative quotations.

### Uncovering the substance facts

This theme incorporates the users’ discussions about specific NPS and their objective properties and appearance. A wide variety of known NPS, such as different types of synthetic cannabinoids, stimulants, dissociatives, and hallucinogens, were discussed. See Table 2 for a full list of NPS found in the thread titles of this study. There were also discussions about branded NPS in which the actual psychoactive component was concealed or unknown, which resulted in elaborate speculations about the content: “*Vendor will not reveal the molecule*”. / “*I have used both chemicals and believe they are different. ‘Sunshine’ is not Methylone either*”. It was very common to compare the NPS discussed with traditional counterpart drugs or previously occurring NPS and rate them accordingly. Another recurring topic was how and where a substance was acquired: “*I really want to try this, could anyone post where to get it?*” to which another user responded: “*Hello, you have an email*”. Selling drugs via the forums or linking to sites that offered drugs was officially not allowed, so these discussions were mostly moderated or continued nonpublicly: “*Can someone pm [Personal Message] me a vendor in the US?*” Different vendors were nevertheless mentioned in talks about their offered products or in discussions related to price, shipping, and the overall evaluation of the vendors’ service. General discussions about NPS availability were also found, which often included descriptions of where the NPS was encountered, such as online or in real life. In addition, the reports of availability were closely linked to discussions of the legal status in different countries: “*Canada’s drug act does not list Ethylphenidate, and no analog law for Methylphenidate exists so you should be legally safe here*”. Potentially legal consequences of possession or purchase of NPS were also mentioned.

**Table 2:** The NPS found among the thread titles

**NPS**	**Discussion thread first started**	**Reported to the EMCDDA for the first time**
AMT, αMT, alpha-methyltryptamine	November 2001	2001
5-MeO-MiPT	September 2003	September 2005
4-FA, 4-fluoroamphetamine	March 2004	December 2008
2C-E	July 2004	Not found
5-MeO-DALT	September 2004	February 2007
4-AcO-DMT	August 2006	August 2009
Methylone	September 2006	March 2005
MDPV, methylenedioxypyrovalerone	October 2006	December 2008
Mephedrone	December 2007	March 2008
HOT-7	March 2008	Not found
Spice (JWH-018)	November 2008	December 2008
3-MeO-PCP	July 2009	March 2012
Methoxetamine	September 2009	November 2010
6-APB, benzo fury	May 2010	June 2011
Ethylphenidate	July 2010	November 2011
25D-NBOMe	September 2010	April 2012
AM-2201	October 2010	January 2011
MPA, methiopropamine	December 2010	Not found
25I-NBOMe	January 2011	June 2012
Allylescaline	February 2011	July 2013
BZ-6378	April 2011	November 2011
a-PVP	November 2011	April 2011
AH-7921	December 2011	August 2012
AL-LAD	December 2011	Not found
2-FMA, 2-fluoromethamphetamine	February 2012	March 2012
MDME	February 2012	Not found
Pyrazolam	July 2012	August 2012
*N*-Ethyl-norketamine	August 2012	September 2012
5-MAPB	December 2012	January 2013
4,4-Dimethylaminorex	May 2013	October 2013

The substances appeared on the forum before reported to the EMCDDA in all except one case. Five substances were not found in the lists published by EMCDDA and therefore not compared.

Other more in-depth substance talk involved chemistry and pharmacology. Users speculated about how substances were built molecularly and how specific chemicals interacted with the brain and body to produce certain effects, for example, “*Both stereoisomers are most likely active (as with amphetamine). In that case, it’s a matter preference*: *dopaminergic or noradrenergic effects. Choose for yourself*”. The origin and history of certain NPS were discussed as well. Furthermore, users talked about how the use of certain chemicals could have contraindications with both traditional substances and other NPS. Related to pharmacology was the topic of tolerance from repeated use of the same substance and cross-tolerance between chemically similar substances. Toxicity was also found to be of interest for the users, and many discussions focused on theoretical speculation of a substance’s overall harm potential: “*The neurotoxicity of 4-Fluoroamphetamine derive from the release of large amounts of serotonin from the neuron, as well as general inhibiting effects of neuronal processes*”. Moreover, discussions focused on the exterior appearance of different NPS and the assessed purity. Users described if the NPS was produced in the form of a pill, powder, or other. The users gave detailed reports on appearance, feel, smell, and taste. They also assessed the identity, quality, and purity from the appearance, for example, “*My powder has a distinct benzene*-*like chemical odor and a blueish/greenish color. Unlike former yellowish odorless batches, I think this one might have impurities*”.

### Dosage and administration

This theme consists of topics related to circumstances surrounding the administration of NPS. Dosage was found to be a widely debated topic of discussion. Many opinions and estimations about dosing and re-dosing were stated. Users also asked for advice on specific dose recommendations with regard to individual traits as previous experience, body weight, built-up tolerance of the drug, etc. They also speculated about how the dose could affect the experience and intensity of effects and suggested different doses for specific purposes, for example, “*I think 15 mg is perfect for moderate effects* - *great for watching movies! Then at 30 mg the visual impressions and euphoria increase dramatically*”. The route of administration was given much attention. Users described several different ways in which different NPS were taken, such as nasally, intravenously, and orally. Opinions about the best way to ingest a specific substance were very common: “*I don’t recommend insufflation and the taste is awful. Rectal administration is recommended for a rapid onset*”. Technical talk about things such as do-it-yourself smoking devices and weighing and purification of a substance were found: “*Did you wash the crystals before recrystallization*? *If so, which solvent(s) did you use?*” Taking a specific NPS in combination with other drugs and how the different effects would interact with or supplement each other were also discussed: “*I want to experience Methylone, Mephedrone and MDPV in one go. Who has tried this or can give advice on dosage?*” The circumstances and surroundings in which the drugs were administered were shared among users. They also asked for, or proposed to others, several settings and recreational activities: “*How would this substance suit a nightclub setting?*” Users also discussed their expectations of the upcoming experience of NPS and their mood or state of mind when taking the drug. They were suggesting and sharing different purposes of using drugs, for example, study aid, recreation, self-medicating, curiosity, and personal development.

### Subjectively experienced effects

This theme was the most prevalent and summarizes discussions about subjective and self-experienced effects induced by a wide variety of NPS. The effects were discussed in great detail and with precision. Many initial or onset effects were described. Also, the primary effects and positive aspects of an experience were shared thoroughly: “*I felt a boost in confidence and was more social, verbal and energized. I was also more curious and present.*” Potency and duration of effects were central and recurring topics in this theme. Specification of the time at which different phases of the experience occurred was mentioned. The discussions of effects were found to consist of physiological, emotional, and cognitive aspects of the experience. Also, users were talking about perceptual and sensory alterations. In addition to the positive effects, many side effects or negative experiences were shared: “*I used this substance for the first time and experienced chest pains and aches all over the body*”. Residual effects or aftereffects were also given attention in the discussions. Users who had not tried a specific NPS asked others to share their experienced effects. There were not many debates or arguments found among the effect discussions; instead, it appeared that the whole community contributed to complete a picture of the overall effects: “*I also experienced that the ‘magic’ of Methoxamine decreased over several successive trips*”. The first posts about experiences with a novel substance were often scientifically depicted: “*T + 0:00 2 mg allergy check intranasally. T + 0:22 Calibration dose 15 mg sublingually. T + 1:10 Subject experiences euphoria equivalent to 40 mg 4-methylephedrone but less stimulation. T + 2:10 Subject confirms a return to baseline.*” References to, and comparisons with, traditional substances and their effects were made to explain the experience to others: “*4-fluoroamphetamine definitely feels like MDMA. The empathy is there and I’m socializing and experiencing a pleasant wellbeing. The stimulation and sharp focus also reminds me of amphetamine*”. In addition, if the NPS in focus had a traditional counterpart substance, it was often used as point of reference to evaluate the effects at large. Rating and evaluation of effects in general were found to be a very common topic: “*I love it! Although it does not have the intensity of MDMA or Mephedrone, it reminds me of their chemicals qualities. This substance is deeper and more multifaceted than amphetamine*”. They also discussed the impact of the experience on everyday life: “*6-MAPB changed my worldview and helped me in a tremendous way*”.

### Support and safety

This theme emphasizes that the discussions involved social support and community help with drug-related issues of concern. Users often asked each other for specific advice on how to prevent or minimize harm. They also expressed the need for support with drug-related problems, to which other users would respond: “*I advise you not to take it under those circumstances because serotonin will be released like with MDMA*”. It was also found that users shared practical tricks and theoretical knowledge with one another to reduce potential problems such as sleep deprivation, contraindications, and the misidentification of substances. Experienced users were asked to verify the authenticity of substances by providing photographs or appearance descriptions. Furthermore, general caution and harm reduction was proposed throughout the forums: “*At this forum we advocate taking a break of at least one month between uses, which applies to 5-MAPB as well*”. Individual users who suspected a dangerous aspect of using a NPS cautioned the whole community. The first users who ordered and used a previously unknown NPS shared their firsthand experience of immediate risks and hazards with the rest of the community. For example, several early warnings about substances or combination of substances that constituted a perceived risk were found: “*Purple Wave of any kind is toxic… do not purchase it*, *you will mess yourself up*”. / “*Be aware of this combo! Some people have had strong reactions to this*”. Less immediate warnings of potential side effects accumulated in the threads of discussion as more users started to report back about the drug: “*I warn you: the residual effects are bad if you binge on it for a while and abruptly stop*”. The abuse potential of different drugs was also discussed: “*In my experience it has highly addictive properties*”. Furthermore, if the first users of an unknown NPS experienced it as safe and reliable, it was reported in the thread as well. Warnings also included notions of bad batches of a substance or scamming vendors.

Discussions were oriented towards acute help and emotional support to users who were online to ask for guidance while intoxicated with fearful or bad reactions: “*Is anyone online because I need assistance with an ongoing anxiety attack?*” Different coping strategies were suggested by fellow users: “*Relax, you will be fine. Put on a movie or occupy yourself by talking to friends.*” Long-term problems like addiction and withdrawal were also topics that emerged. Users asked about abuse problems, and others were being supportive or offered their experience on how to solve them. Also, several recovery reports were shared publicly to other users, who responded with encouraging comments or grateful remarks.

## Discussion

This study has explored and characterized the discussions about NPS on international Internet forums. The most post-frequent discussion threads from three “leading edge” international Internet forums were systematically analyzed for recurring topics. Fifty-one categories of discussion emerged, which were combined into four overarching themes that characterized the discussions: (1) *uncovering the substance facts*, (2) *dosage and administration*, (3) *subjectively experienced effects*, and (4) *support and safety*. The findings indicate that the discussions involved an extensive exchange of knowledge about NPS and how to use them safely. The discussions were characterized by a communal process in which forum users supported each other and contributed with cumulative experiences and knowledge whenever a new thread of discussion brought awareness to a previously unfamiliar substance of interest. More specifically, the initial and continuous characteristic of this generative process was to *uncover the substance facts*, which incorporated elaborate theoretical speculation of origin, classification, and molecular structure, as well as pharmacological action, tolerance, and toxicity. Many users displayed a very high level of knowledge, which reflects a dedicated interest in NPS as well as a focus on preparation and safety. The accuracy of single posts of information is difficult to assess since reliable knowledge about these substances is scarce. The general principles of toxicology and pharmacology were however appropriately followed. More interesting is that every post contributed to a greater body of cumulative community knowledge which could be publicly assessed and continuously refined by anyone on the forum. Another interesting finding is that discussions occasionally focused on revealing the concealed or unknown psychoactive constituents in branded NPS with content specification intentionally left out. When possible, the actual appearance was used for identification and assessment of quality. As information about a NPS increased, and comparisons with similar types of substances were made, an overall assessment of expected effects and potential risks and benefits started to form. By then, the users also discussed both the legal status in different parts of the world and the availability and acquisition of the NPS. In addition, different vendors were evaluated for their customer service and quality of product, indicating the awareness of safe drug use.

As the uncovering of facts progressed, expectancies of the actual NPS started to emerge, and the process of information exchange was further characterized by an added focus on *dosage and administration*. In short, users discussed virtually everything that was related to the knowledge of getting the drug into the body. Personal preferences and different individual conditions gave rise to many opinions and suggestions. Furthermore, effect- and purpose-specific dosing and method of administration were thoroughly requested and provided by users, so were different sets and settings in which the use of drugs occurred, as well as do-it-yourself techniques for enhancement of administration. Discussions about ingesting a combination of drugs for supplementary and interactive effects were also frequently discussed. The majority of these topics correspond to discussion characteristics found among a sample of online ecstasy users [30], which not only add support to the present study but also indicate that the result apply, at least partly, to online drug discussions in general.

Gradually, numerous and very detailed descriptions of *subjectively experienced effects* started to characterize the discussions, which was interpreted as an expression of a substance’s increased availability and popularity over time. Self-reports of firsthand experienced effects were very common and constituted the most prevalent theme. A whole spectrum of effects, as well as potency and duration, were depicted and shared with the community in a precise and technical manner. The connoisseur-like accounts of experience contributed to the value of using Internet discussions as a source of detailed data for scientific studies where triangulation is required or relevant data are scarce. Users were inclined to review and rate their experience, as well as compare it with effects of the NPS’s traditional counterpart drug. In addition, the descriptions of experiences were cumulative and appeared to generate a consensual evaluation of a substance’s usefulness. This might help explain why some NPS become more popular and widely spread than others. Interestingly enough, the users emphasized both positive and negative experiences of effects, which indicate that the reports were retold with a thorough and comprehensive ambition. In earlier studies based on self-reported data [21-23], it was suggested that experiencing per se was more important than the actual content of the experience (fear or euphoria), which offers an explanation for the high prevalence of nuanced and balanced descriptions.

The findings in the present study provide an additional explanation to the absence of one-sidedly positive and drug romanticizing discussions, namely that users were concerned with the *support and safety* of themselves and others. The result showed that the discussions were served as a user-governed early warning system where, for example, acute risks and side effects were posted whenever a dangerous or unhealthy aspect of a NPS was encountered. Furthermore, the discussions were characterized as a support system where different types of individual concerns were responded to with advice, practical tricks, and knowledge in order to prevent and minimize harm. In fact, *harm reduction* appeared both explicitly and implicitly as the common denominator that on the whole permeated the discussions. At a higher level of abstraction, *harm reduction* emerged as a characteristic that was present in all themes in this study. The significance of harm reduction is further supported by the findings in previous studies where drug-related discussion forums have been examined [27,28,30]. However, publicly available discussions have at least two conceivable flip sides. First, Barratt et al. [31] have revealed a less acknowledged and oppositional discourse to harm reduction which incorporates a desire for dangerous drug use and downplay of harm reduction. This alternative discourse was not revealed by the present study. Nonetheless, the present study exposed another possible flip side: uncovering the substance’s facts will, apart from reducing harm by minimizing uncertainty and unpredictability, potentially result in publicly available knowledge about, e.g., acquisition of a drug. Even though the risk of increased substance use exists, evidence [32] indicates that exposure to drug information does not have counterproductive effects. Nevertheless, this dilemma highlights the previously debated issue [28,33] whether the focus on prevention should be a reduction in overall use or harm. In any case, the present study showed that the extensive and cumulative process of knowledge exchange and social support enabled by Internet discussion forums offers valuable insight of use to prevention strategies aiming at a reduction of harm. All except one of the substances found in the thread titles appeared as an online discussion before reported to the EMCDDA (see Table 2). The forums could therefore be used for quicker monitoring and risk profiling. Internet forums are not only an undervalued location for drug prevention; further research should take greater advantage of the publicly available information to investigate new drugs, their effects, and the motivation for using them.

## Conclusions

Discussions on drug-related Internet forums were characterized by a communal process in which users supported each other and exchanged an extensive and cumulative amount of knowledge about previously unfamiliar substances. The discussions uncovered the substance facts such as identity, origin, quality, legal status, acquisition, and pharmacology. Users also talked about circumstances related to the administration, including intentions, settings, individual dosing, safe combination of drugs, and administration techniques. Furthermore, a plethora of subjectively experienced effects were shared among users in a connoisseur-like manner. These reports were nuanced and comprehensive, which was suggested to reflect the users’ concern for support and safety. On the whole, harm reduction emerged as the main characteristic that permeated the discussions. In addition, users warned each other about potential risks and side effects and supported each other with advice and guidance. The findings in the present study contribute to the understanding of the online reality that faces youth in search of drug-related information. Also, the discussion forums could be used for drug prevention, as well as a source of information for further research about NPS.

## Competing interests

The authors declare that they have no competing interests.

## Authors’ contributions

AK and CS conceived and designed the study, analyzed the data, contributed to the writing of the manuscript, and agreed with the manuscript results and conclusions. CS searched and collected the data from the forums. Both authors reviewed and approved the final manuscript.
